# Recent Development in Biomedical Applications of Oligonucleotides with Triplex-Forming Ability

**DOI:** 10.3390/polym15040858

**Published:** 2023-02-09

**Authors:** Incherah Bekkouche, Alexander Y. Shishonin, Alexandre A. Vetcher

**Affiliations:** 1 Nanotechnology Scientific and Educational Center, Institute of Biochemical Technology and Nanotechnology, Peoples’ Friendship University of Russia (RUDN), Miklukho-Maklaya Str. 6, Moscow 117198, Russia; 2 Complementary and Integrative Health Clinic of Dr. Shishonin, 5, Yasnogorskaya Str., Moscow 117588, Russia

**Keywords:** oligonucleotides, triplex, nucleic acids, pH, aptamer, biosensor

## Abstract

A DNA structure, known as triple-stranded DNA, is made up of three oligonucleotide chains that wind around one another to form a triple helix (TFO). Hoogsteen base pairing describes how triple-stranded DNA may be built at certain conditions by the attachment of the third strand to an RNA, PNA, or DNA, which might all be employed as oligonucleotide chains. In each of these situations, the oligonucleotides can be employed as an anchor, in conjunction with a specific bioactive chemical, or as a messenger that enables switching between transcription and replication through the triplex-forming zone. These data are also considered since various illnesses have been linked to the expansion of triplex-prone sequences. In light of metabolic acidosis and associated symptoms, some consideration is given to the impact of several low-molecular-weight compounds, including pH on triplex production in vivo. The review is focused on the development of biomedical oligonucleotides with triplexes.

## 1. Introduction

**Figure 1:**
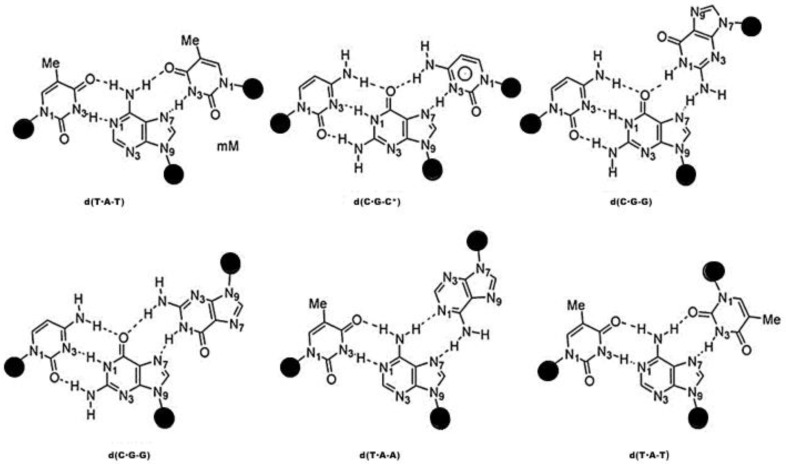
Hoogsteen-based triads are shown schematically in parallel and anti-parallel triplexes, as well as in triads based on the reverse Hoogsteen algorithm.

Triplexes are non-canonical DNA structures that contain an extra single-stranded RNA or DNA binding region that is unique to the primary groove of double-stranded DNA. Triplex-forming oligonucleotides (TFOs) bind in the duplex major groove and produce a triple-helical structure. By creating hydrogen bonds with exposed groups on Watson-Crick base pairs, substances that directly interact with duplex DNA are of interest as gene targeting techniques. Research on these substances has centered on the use of triplex-forming oligonucleotides (TFOs), which interact with one another from the major groove side of duplex DNA to generate triplex DNA in a sequence-specific way [1,2]. The ability to inactivate and activate gene expression, recombination, and repair through the production of triplex DNA make this technique potentially useful for analyzing activities involving the genome [3,4,5]. With every sequence of duplex DNA, the ability to generate stable triplex DNA is, however, inherently constrained. With guanine at the GC base pair and adenine at the AT base pair from the main groove side of the target duplex DNA, respectively, guanine and adenine nucleobases in TFOs rich in purines create two reverse Hoogsteen-type hydrogen bonds (Figure 1) (e.g., [6]). The formation of stable triplex DNA using TFOs made of natural nucleosides is not conceivable, though, since the CG and TA base pairs only have one hydrogen bonding site at the main groove side of the cytosine and thymine nucleobases.

Triplex technology still has some flaws [7], most of which are related to (i) their low stability, (ii) sequence restrictions due to the requirement of polypurine tracks in the triplex target sequence, (iii) susceptibility to nucleases, and (iv) difficulties in delivering TFOs in the cellular nucleus. A substantial amount of chemical, biochemical, and biotechnological research is presently being directed toward resolving the practical issues associated with triplex technology [8,9,10,11]. In this study, we conduct a thorough examination of the most recent advances in biological applications of oligonucleotides with triplex-forming capacity.

## 2. Results and Discussion

### 2.1. Oligonucleotides with Triplex-Forming Ability in Targeted Delivery

Genomic DNA is frequently under torsional stress, which can cause the DNA double helix to be both over- and underwound. A decrease in the number of links (Lk) connecting the two strands of a closed-circular DNA (a negative Lk) causes negative superhelical stress. The untwisting of the helix (change in twist; Tw) and a coiling distortion of the DNA backbone (writhe; Wr) are the two components of the conformational response to this stress, known as negative supercoiling [12,13,14,15]. Genomic DNA in prokaryotes supercoils with an average density of (Lk/original Lk) of 0.065. Nuclear-associated proteins and supercoiling collaborate to control bacterial gene expression [16]. Supercoiling produced by transcription in eukaryotes is thought to be involved in the control of oncogenes such as c-Myc. It affects the positioning of RNA guide sequences by the CRISPR-Cas9 gene editing toolkit [17] and plays a crucial role in the development and stability of looped DNA structures [18] and DNA R-loops [19].

#### 2.1.1. Substantial RNA–DNA Interactions

RNAs have been assigned many regulatory roles, some of which involve interactions with both DNA and proteins [20,21]. The addressing and recruitment of transcription factors [22,23], the structuring of the transcription factor machinery [22,24], and the mediating of histone modifications are all tasks performed at the RNA–protein interface. The functional role of the XIST transcript in the silencing of the X chromosome during dosage compensation is just one example of the epigenomic effects associated with RNA–DNA interactions [25]. G-quadruplex [26] creation is another further instance of RNA–DNA interactions. R-loop [27]. R-loops, which are associated with chromatin condensation and cancer, are interactions between single-stranded DNA and RNA that are made possible by Watson-Crick base pairing [28,29]. In addition to these, there is another type of RNA–DNA interaction in which the double helix’s primary groove is occupied by single-stranded RNA, maintaining the double helix’s structural integrity, which results in the development of a triple helix (triplex) comprising RNA and DNA. triplex production, is an aspect of epigenetics that, despite long-standing knowledge in the biophysical sense [30,31], is still not fully understood this is due to a few factors, primary among which is the difficulty of conducting experiments to investigate triplex formation on a genome-wide level in living cells. In the past, approaches for capturing the genomic interaction locations of individual transcripts have been used to experimentally investigate triplex formation in the setting of cells [32,33]. However, across a wide range of species, tissues, and cell types, regulatory transcripts have been connected to epigenetic processes [34,35,36,37]. Herein lies the significance of creating instruments that can precisely forecast RNA–DNA interaction points as possible sites of triplex formation. Hoogsteen base pairing rules [38], which are canonically accountable for triplex formation, have been used as the basis for previously reported computational tools (Triplexator/Triplex Domain Finder [39,40], as well as LongTarget [41]). These are tools for applying Hoogsteen rules. A comparison of Triplexator and LongTarget utilizing MEG3 ChOP-seq data [32] shows significant space for improvement in this area, even though the use of canonical rules to predict triplex formation offers insight into potential RNA–DNA interactions [42]. These benchmarking data imply that base pairing rules that go beyond the Hoogsteen base pairing rules now in use may be necessary to implement reliable prediction of triplex formation in a cellular context. In relation to this, research has been conducted on the prediction of triplex-forming RNA and DNA sequences using previously documented triplex interactions [43], albeit this method cannot predict entire triplex interactions. Experiments with comparable goals have been used in conjunction with computational methods to anticipate RNA–DNA interactions across the entire genome. In terms of triplex creation, the genome-wide isolation of triplexes, followed by sequencing (triplex seq), are the most important of these [44]. This technique enables the identification of triplex-forming sequences throughout the genome (triplexDNA-seq) and transcriptome (triplexRNA-seq), but it does not provide information on how the sequences couple up with one another. Numerous approaches have been described to find all to all contacts between transcripts and chromatin aside from specifically triplex-mediated RNA–DNA interactions [45,46,47,48]. However, RNA and DNA interacting complexes ligated and sequenced (RAD-ICL-seq) [49] and RedC [50] have nucleotide processing processes that are the most similar to those of triplex-seq. Through the proximity-based ligation of RNA and DNA via a linker sequence, these approaches together discover relationships between transcripts and areas of the genome. Although they are still relatively new and experimentally challenging, RADICL-seq and RedC are valuable sources of information on RNA–DNA interaction. Therefore, it is not now viable to conduct such studies under a variety of steady-state and differential situations. As a result, the most broadly applicable use for this data may be as inputs for machine learning algorithms, which could enable the prediction of RNA–DNA interactions under a condition of interest.

A type of direct RNA–DNA interaction mechanism known as a triplex is created by the binding of RNA sites to a purine-rich strand of duplex DNA in accordance with either the forward or reverse Hoogsteen base-pairing rule. It has been established that some lncRNAs create DNA to carry out certain functions. For instance, promoter-associated lncRNA interacts with TTF-I to inhibit the transcription of rRNA [51], and FENDRR increases PRC2 occupancy at the triplex formation sites [52]. MEG3 forms a DNA-lncRNA triplex with the TGF gene to regulate the activity of the gene [32], and the particle binds to the MAT2A promoter CpG island as a triplex to support gene-silencing mechanisms [53]. KHPS1 and SPHK1 interact to bind the lncRNA and associated effector proteins to the gene promoter [54]. HOTAIR and PCDH7 and HOXB2 form a triplex to control adipogenic differentiation [55]. MIR100HG controls p27 via the formation of a triplex [56], and the promoter and pre-rRNA antisense direct associated CHD4/NuRD to the rDNA promoter [57] chromatin isolation by RNA purification (ChIRP-seq) [33]. Capture hybridization analysis of RNA targets (CHART-seq) [58], RNA antisense purification (RAP-seq) [59], and chromatin oligo affinity precipitation (ChOP-seq) [52] are examples of recently developed high-throughput techniques that have assisted in creating the genome-wide map of lncRNA chromatin interactions for specific ones. As a result, they are unable to offer trustworthy sources for research into DNA: RNA triplex production. Cetin et al. [44] devised a method to map the genome-wide DNA: RNA triplexes by eliminating the chromatin crosslinking to demonstrate the existence of the DNA: RNA triplex interactions in vivo. The physiological significance of DNA: RNA triplex complexes was demonstrated using this method. Currently, base paring rules related to mathematical statistics are primarily used to predict DNA: RNA triplex. Hoogsteen and reverse-Hoogsteen base-pairing are taken into consideration in the proposed triplexator to systematically discover the probable triplex formation sites of RNA and the targeting sites on DNA [39]. Triplex-Inspector was created to choose sequence-specific ligands and targets while taking genomic architecture and gene location into account [60]. A long target was created to find motifs and binding sites in triplex formation while taking non-canonical rules into account [41], and triplex Domain Finder (TDF) was created to predict triplexes and to characterize lncRNA and the corresponding DNA targets [40].

In several biologically significant processes, including DNA replication, transcription, telomere replication, and HIV replication by reverse transcription [61,62,63,64,65], DNA: RNA hybrids are generated as intermediary structures. Additionally, they have been found to form R-loops in a variety of organisms, including bacteria and humans, and these loops are essential for controlling gene expression, DNA and histone modifications, immunoglobulin class switch recombination, DNA replication, and genome stability [66]. Small organic compounds having the capacity to specifically restrict DNA replication via Okazaki fragments, therefore also preventing transcription, offer a great deal of promise in the treatment of cancer because the replication is frequently enhanced in cancer cells furthermore, telomerase and RNase inhibitors, which are tiny compounds selective for hybrid duplexes, may be used therapeutically [67,68,69,70].

The discovery of substances that specifically attach to DNA: RNA hybrids have only been briefly discussed in the literature [71,72,73]. The existence of a few ligands that selectively bind DNA: RNA hybrids are shown by the literature sources [71,72,73,74,75,76]. A common structural motif that preferentially binds to the hybrid structures was discovered in extensive research by Arya and Chaires using quick screening assays, competition dialysis, and thermal denaturation of mixtures [71,72,73,74,75,76,77]. Ethidium bromide, coralyne, amino glycoside, propidium, thiazole orange, and ellipticine, among others, showed preferential binding to hybrid duplexes among other nucleic acid structures. These substances all possess the common motif planar aromatic ring system with a “bay” area. Triplex structures have been discovered in biologically important RNA molecules, such as telomerase RNAs, [78,79], long noncoding RNAs (lncRNAs) [80,81], RNA pseudoknots responsible for 1 programmed ribosomal frameshifting (PRF) [82], catalytic centers of spliceosomes [83], bacterial SAM-II riboswitch [1], or self-splicing group I [84].

Given the importance of triplexes in RNA biology, tiny compounds capable of recognizing, binding, and stabilizing triple helical RNA structures are gaining attention as potential molecular biology tools and therapeutic agents in contrast to many investigations on DNA triplexes [85,86]. The interaction of small molecules with RNA triplexes has received significantly less attention than organic compounds, aminoglycosides, and various dyes, which are examples of compounds that stabilize RNA triplexes, such as neomycin [87] tunable diphenylfuran based scaffold (furamidine) [88], imidazoles, benzimidazoles [89], flavonoids [90], alkaloids, and their analogs [91,92]. Proflavine and its conjugate [93] and transition metal [94,95,96] intercalation and groove binding are the key mechanisms by which these chemicals interact with RNA triplexes.

#### 2.1.2. PNA

Noncoding RNA (ncRNA) participates in crucial but poorly understood biological processes, and the emergence of illness [97,98,99] research tools for sequence-specific recognition, detection, and functional inhibition of such RNAs would be very beneficial for both theoretical applications in biology and real-world biotechnology and medical applications. A lot of non-coding RNAs acquire complicated tertiary structures with double helical sections that can be targeted via triple helical recognition. The molecular identification of such helical RNA structures has not, however, received much research [100,101].

Triplex forming oligonucleotides (TFOs), or their chemically modified counterparts, can identify double-helical DNA and RNA in a sequence-specific manner [86], but a significant obstacle to the synthesis of triple helices is that only the purine nucleobases can be identified through two Hoogsteen hydrogen bonds in the naturally occurring T*A-T (or U*A-U) and C + *G-C triplets. Because inverted U-A and C-G base pairs only have one hydrogen bond donor or acceptor in the main groove of the duplex, it is extremely difficult to recognize pyrimidine nucleobases in these base pairs. Due to the electrostatic attraction between a TFO’s negatively charged phosphates and the target nucleic acid duplex, low binding affinity and sluggish kinetics of TFOs are also issues, as is the requirement for protonation of cytosine (pKa 4.5) to generate the unfavorable C + *G-C triplet. In recent developments of cationic triplex-forming peptide nucleic acid, the latter two issues have been resolved (PNA) [102,103,104,105,106,107]. The nucleobases of PNA are joined by a pseudopeptide backbone, making it a neutral DNA counterpart. PNA was first designed as a double-stranded DNA (dsDNA) triplex-forming ligand [108]. However, it was later shown that PNA can invade dsDNA by dislodging a pyrimidine rich DNA strand and generating a PNA-DNA-PNA triplex, opening a brand new and fascinating mode of DNA recognition. Although triple-helical PNA binding to dsDNA has also been investigated, the first report of PNA binding to dsRNA was made by Rozners and colleagues in 2010 [109,110,111,112,113], along with others [104], who demonstrated that dsRNA and nucleobase-modified PNA produced triple helices with great affinity and sequence specificity [109,110,111,112,113].

PNA-dsRNA triplex production at physiological pH and salt concentration was made possible by swapping out the more basic cytosine nucleobase for the more basic 2-aminopyridine (M) nucleobase (pKa 6.7, hence partially protonated at physiological circumstances). Building on the work of Corey [114,115] and Gait [116,117], conjugation with cationic lysine residues improved the binding affinity and accelerated cellular absorption of the cationic triplex forming PNA without impairing the binding specificity [106,107]. The most striking finding was that PNA exhibited an approximately 10-fold greater affinity for complementary dsRNA than for the same dsDNA sequence. However, sequences with most purine nucleobases on one strand of the dsRNA were still required for the creation of extremely stable PNA-dsRNA triplexes (polypurine tracts) [105,106,107].

One may argue that the most important restriction on triple helical identification of nucleic acids is the requirement for polypurine tracts [118]. A few research teams have created heterocyclic nucleobases that can join the exocyclic -NH2 of cytosine to form a single hydrogen bond. Leumann demonstrated that the C–G inversion was preferentially recognized by 5-methylpyrimidin-2-one (4HT, R2 = CH3) modified TFOs over other base pairs, but the binding affinity was lower than that of conventional Hoogsteen triplets [119] when pyrimidin-2-one (P, R2 = H) was added to triplex forming PNA targeting dsRNA [120] despite having a decreased affinity, as well as P-modified PNA still bound to mRNA in a triplex with a hairpin structure, which prevented it from being translated both in vitro and in living cells [118]. The latter investigation was the initial proof of the biological impact of PNA-dsRNA triplex production in living cells [121].

A tried-and-true technique for low-cost, high throughput relative quantification is the use of DNA microarrays these arrays are used for quick screening of up/down-regulation. However, they have a limited dynamic range, and their ability to hybridize is heavily dependent on factors, including temperature, ionic strength, and probe affinity [122]. Another well-known technique, qRT-PCR, provides for the accurate quantification of numerous miRs and is frequently used to validate the results of microarray analysis on a particular collection of targets. Finally, RNA sequencing is a well-known technology used for the accurate identification and quantification of miR sequences, as well as for the discrimination of closely related sequences.

Some drawbacks to PNA use should be considered while designing probes. Reduced solubility in aqueous media is a side effect of the neutral poly-amidic backbone, which ensures the production of robust duplexes with natural nucleic acid targets. By adding charged and hydrophilic residues, such as charged amino acids, this can be easily prevented (i.e., arginine, glutamic acid, etc.) when operating at the low micromolar concentrations typically used for oligonucleotide detection, this lowered solubility limitation is typically less significant, but it could still be a problem when it comes to the manufacture of the device. For instance, it has been demonstrated that the polyamidic backbone of PNA causes issues when thiolated probes are conjugated to the surface of gold nanoparticles [123], and to address this, alternate gold decorating protocols must be developed [124,125]. Furthermore, enzymes, such as ligases or polymerases, which are currently used for amplification-based detection methods depending on DNA probes, are unable to recognize PNAs due to their artificial origin. PNAs have found many uses in nucleic acid-targeting strategies and have been proposed as biomolecule-based drugs and as probes for diagnostic tools [126,127,128,129]. Despite these drawbacks, which may deter their application, given their straightforward conjugation with peptide sequences and small functional groups and the benefits in terms of duplex stability, PNAs have found a variety of applications in these fields. The objective of this review is to provide a summary of current advancements in PNA-based miR detection methods, paying particular focus to the key advantages over conventional DNA-based approaches.

### 2.2. The Impact of TFOs on Gene Expression

The sequence of RNA and DNA defines its secondary and tertiary structure. Their affinity and specificity are finally defined by the structure [130]. Single stranded DNA or RNA molecules are purpose-built to attach to a certain target molecule. As a result, they might be used as components of macromolecular devices for basic research and technological applications. Because of interactions with complementary portions of the chain and stacking, each aptamer has a distinct three-dimensional structure.

The in-cell NMR approach has been proven to be a reliable tool for determining the intracellular structures of oligodeoxynucleotides. Several groups, including ours, have used in-cell NMR to directly identify the production of DNA hairpins, [131] RNA hairpins, I-motifs [132], G-quadruplexes [133], and Z-DNA [134] structures in living human cells. The NMR approach was also used to assess tRNA changes in real-time in yeast cell lysate [135,136,137].

According to research, approximately 97% of human genes have at least one triplex forming sequence, with 86% having a unique arrangement [88]. Because of this, as well as their programmability and selectivity, the creation of efficient TFOs is an essential problem because it gives a mechanism to directly control gene expression [138] with further applications in bionanotechnology and synthetic biology [1].

#### Triplex-Mediated Modulation of Gene Transcription

A multistep process called transcription involves the dynamic interaction of DNA with various protein partners as well as the synthesis of DNA-protein complexes and structures with various inherent stabilities. It has long been believed that it is simpler to inhibit RNA synthesis during transcription elongation than to negatively (or positively) modulate transcription initiation via triplex competition with transcription activators (or repressors) [139].

### 2.3. Biosensor Based on Aptamers

Aptamer based biosensor designs have received a lot of interest in the disciplines of biotechnology and chemistry [140,141,142,143]. Single stranded RNA or DNA oligonucleotide aptamers with distinct intramolecular structures have been utilized to influence chemical production, and their selectivity and affinity for target have been used in various types of biosensors [144,145]. Aptamers are usually intended to recognize and bind to the target when paired with a biosensor to create an aptasensor, with the conformational change employed to generate many detectable signals. Aptasensors have frequently been used to test mycotoxins because of their capacity to concurrently measure bind constants for most mycotoxins, as well as dissociation constants ranging within the nanomole [146,147,148,149]. Wu et al., for example, created a one-step electrochemical aptasensor for the assessment of OTA utilizing a thiol and methylene blue dual-labeled aptamer-modified gold electrode and demonstrated a detection limit of 0.095 pg/mL [150]. Lv et al. reported a fluorescence test for OTA detection based on aptamers and gold nanoparticles with a detection limit of 5 nM [151]. Lin et al. created a simple, low-cost, and sensitive liposome-based colorimetric aptasensor to detect OTA, with a detection limit of 0.023 ng/mL [152]. A triple helix aptamer probe (TAP) was introduced as a biosensing strategy, with critical reference to Watson-Crick and Hoogsteen base pairings. The probe consisted of an intended aptamer sequence with two arm-like segments flanked by the sides of the aptamer and a triplex forming oligonucleotide [153] This sensor offered various benefits over traditional duplex DNA biosensors. Aside from its duplex DNA-like stability, the tap was painstakingly developed with a longer aptamer sequence within the loop, strengthening selectivity, binding affinity, and sensitivity to the targets [154,155,156]. Furthermore, the aptamer could be changed without having to modify the probes for signal transduction, allowing it to be used to detect a variety of targets. Because of these advantages, the tap has received increased interest from scientists and has been used in a variety of innovative biosensing technologies.

### 2.4. G-Quadruplex Forming Aptamer

**Figure 2:**
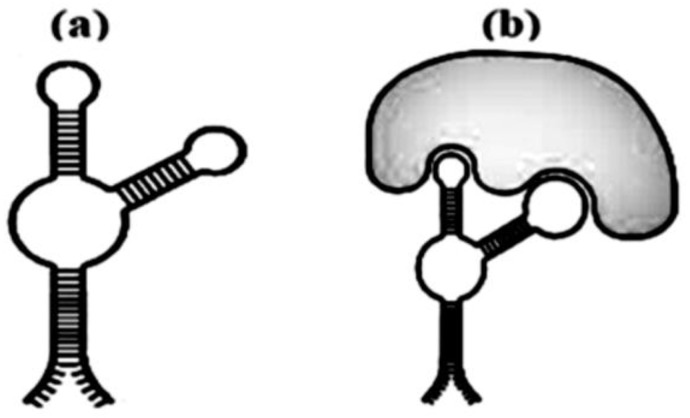
Aptamer secondary structure (**a**) and aptamer with a marker (**b**).

The finding is related to nanobiotechnology and molecular medicine and involves DNA aptamers to the thrombin exosite I interact with prothrombin. These chemicals have the potential to be exploited to develop treatments that prevent intravascular thrombus development. Aptamer-based biosensors for sensing lateral flow are described here [157]. Aptamers are extremely valuable and include biorecognizing chemicals for the construction of biosensors. Aptamers are single-stranded strands of DNA that are used to select protein targets. SELEX is a selection approach that permits the separation of motivated nucleic acid molecules from a huge collection (more than 1015) of individual molecules, known as a combinatorial library [158,159]. Some of the aptamers in Figure 2 (modified by authors from [160]) have a good probability of not only identifying their targets, but also of destroying their biological vitality.

The SELEX technique is used to find and select aptamers. It happens because of exponential enrichment in the systematic development of ligands. The oligonucleotide library is gradually enriched with sequences that have a higher affinity for the target molecule. The process of producing target incubation, elution, and amplification of the binding sequence is continued in classical SELEX [161] until the great majority of the stored pool consists of target binding sequences.

### 2.5. Nucleic Acid Motifs with pH Sensitivity

**Figure 3:**
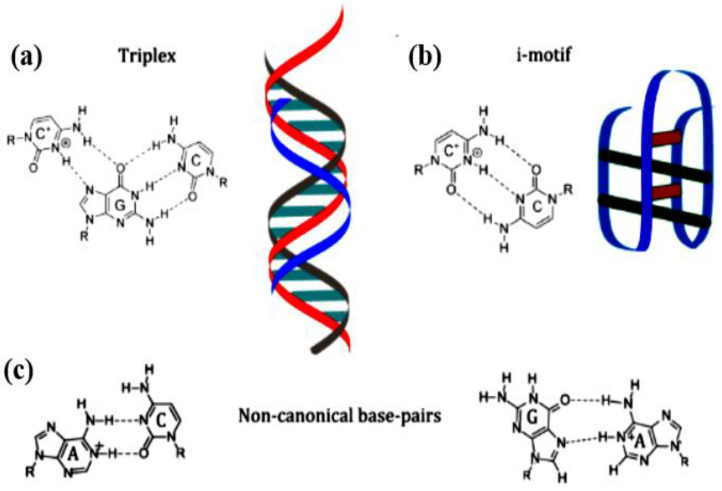
Nucleic acid base-pair binding and motifs that respond to pH. Illustration of the triplex binding motif and C + GC triplex non-canonical base pair binding in (**a**). Illustration of the i-motif, which is made up of C + C bonds, and non-canonical C + C base pair binding in (**b**). (**c**) Left: A non-canonical base pair binding is shown. Right: Illustration of A + G non-canonical base pair binding.

Many pH-responsive motifs have been discovered in DNA structure research that may be carefully tailored to achieve conformation flipping at a chosen pH value (see Figure 3) [162]. Other than the classic Watson-Crick orientation, nucleic acids can attach in a variety of orientations, including reverse Watson-Crick, Hoogsteen, and “wobble” base pairs. Many of these interactions may be found in aptamer structures. The G-quartet motif, for example, is created by Hoogsteen interactions between guanines from four different strands, with each guanine at right angles to the next on a linear plane [163] (Figure 3). When numerous G-quartets (typically three) are layered, the resultant helical structure is known as a G-quadruplex [164]. G-quadruplexes are often found in aptamers and other synthetic DNA nanostructures, as well as mRNA and gene promoters [164]. Protonation of nucleobases can considerably enhance the stability of some base pair mismatches [163]. Adenine and cytosine, in particular, have been seen to become protonated at moderately acidic pH depending on the structure of the folded nucleic acid. The pKa of protonation can be adjusted up to neutrality, allowing mispairs to be stably absorbed into a structure [165,166]. Because protonation allows various base pairs to become stable, altering the pH of the environment can allow a DNA structure, such as an aptamer, to change conformation reversibly [163]. This conformational shift influences features, such as target binding.

The I-motif is a frequent pH-responsive DNA motif. The I-motif is made up of two intercalated duplexes connected by antiparallel C + C base pairing [167]. Isolated cytosine has a pKa of 4.58 [168] at neutral pH. I-motifs can develop due to crowding or super helicity. A new study suggests that this structure affects replication and transcription in vivo [169]. I-motifs, on the other hand, are completely folded at pH 5–6 and unstable at neutral pH under most in vitro circumstances [168,169]. Because of its pH sensitivity, the I-motif has become popular for nanodevices engineered to flip conformation under moderately acidic circumstances [168]. Bielecka and Juskowiak developed a fluorescent probe for pH monitoring based on I-motif creation in 2015, modifying the protonation equilibrium of a fluorescent cytosine analog and thereby quenching it under more acidic circumstances [170]. I-motifs have also been used in more complicated nanostructures, such as logic gates and motors [162]. Two aptamers that employ the I-motif to integrate pH-dependent binding are mentioned below.

A second common DNA pattern that reacts to pH is the triplex structure, several base combinations can produce a triplex, which consists of two strands in a Watson-Crick duplex and a third, extra strand connected to one of the other two through Hoogsteen or reverse Hoogsteen interactions [171]. The triplex motif has been utilized to generate structural change depending on a variety of triggers, the most prevalent of which is strand displacement [170]. However, changes in pH can be utilized to maintain or disrupt a triplex: the C + GC triplex motif, for example, is dependent on the protonation of the cytosine on the third strand [171,172].

The possible pH range for triplex DNA production can be varied by using different triplex DNA sequences. Idili et al. disclosed a logical design of a triplex DNA structure with varying quantities of T A T/C G triplets that “opened” or “closed” based on an intramolecular triplex structure [173]. In addition to constructing a pH-responsive sensor with a large pH window, they were also able to fine-tune the pH below which the switch was closed by altering the percentage of C + GC triplets (pKa 4.5) and TAT triplets (pKa 10.2). These are two aptamers that govern target binding via pH-controlled triplex synthesis. The A-motif, a parallel duplex comprised of A + A + base pairs that develop at pH 3–4, is another pH-responsive motif. Because the motif is more acidic than the I-motif or triplex, it has been proposed that the A-motif may be beneficial for developing pH-sensitive probes below the 5.5–7.6 range, most easily identified by I-motif probes [174]. Other non-canonical base pairs, in addition to the motifs mentioned above, can be employed to introduce pH-controlled aptamer binding when protonated, A + G, A + C, and C + C base pairs all become substantially more stable [173].

### 2.6. Triplex Cations

For single-molecule force spectroscopy, a rescue rope technique was created that allowed us to repeatedly alter the structures generated at free ends of DNA, such as triplex DNA, telomeric DNA, or broken dsDNA following click chemistry conjugation. DNA of interest split apart from the rope DNA handle. The rescue rope DNA assumed a shaped conformation, and DNA of interest creates structures at the crossing point, according to AFM imaging. Further proof that the DNA of interest may repeatedly form structures after being ruptured by stresses came from single-molecule magnetic tweezers. It has been also shown how to mechanically probe DNA triplexes using the rescue rope approach. DNA triplexes can be either YR*Y or YR*R (Y for pyrimidine and R for purine) arrangements, depending on whether the TFOs attach to the dsDNA’s purine strand via forward or reverse Hoogsteen pairings (*) in YR*Y or YR*R triplexes. The mutual orientation of the chemically homologous strands might be either parallel or antiparallel. It has long been suggested that a telomeric triplex model in a YR*R arrangement prevents digestion and recombination with a dsDNA of (TTGGGG)_3_ and a 3 overhang of (TTGGGG)_2_. A DNA triplex occurs in a small tetrahymena telomere, where the G rich overhang folds back and resides in the main groove of the duplex by generating the CG*G triads in an antiparallel YR*R configuration [175].

**Figure 4:**
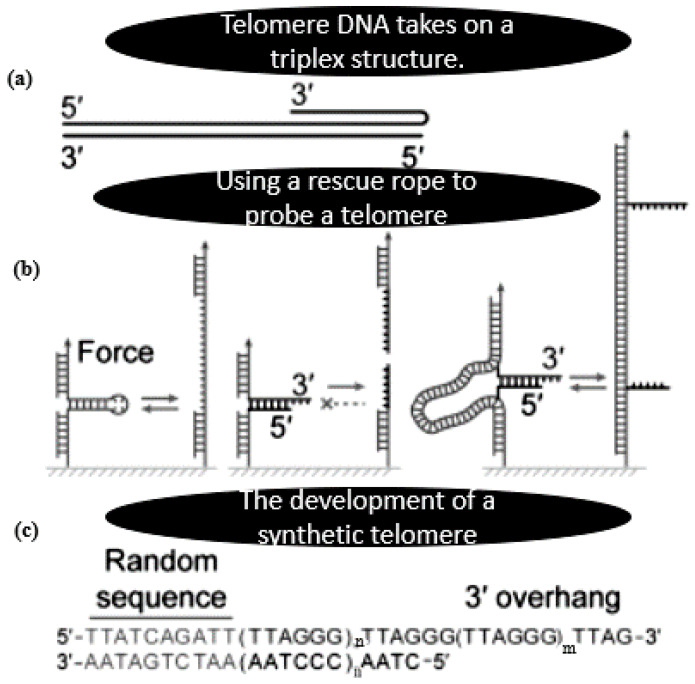
A rescue rope approach for investigating an artificial telomere. (**a**) The DNA in telomeres adopts a triplex shape. The G-rich and C-rich strands are denoted by red and black, respectively. (**b**) Using a rescue rope to probe a telomere with a free end. Structures having a closed end can be repeatedly manipulated by conventional mechanical pulling experiments, such as a DNA hairpin (left). A construction with an open end cannot rotate mechanically in circles before relaxing (middle). A rescue-rope technique supported by dsDNA enables recurrent operations on a structure with a free end, such as a telomere (right). (**c**). Designing a synthetic telomere. The identical configuration of the artificial telomere in melting/reannealing rings is guaranteed by a random sequence at the blunt upstream end. In a duplex and a three overhang, the TTAGGG motif count might change. With n = 5 and m = 2.

Using rescue rope strategy-assisted single molecule force spectroscopy, the production of triplexes by a short human telomeric DNA with eight repetitions of TTAGGG in the dsDNA and the ssDNA overhang has been looked at. The findings showed that the three overhangs of TTAGGG, which are most likely formed by CG*G and TA*T triads, can occasionally fold back to bind dsDNA and create mechanically stable triplexes. The interactions between the overhang and the dsDNA may change because of the TTAGGG repeating motif, resulting in a folding turn of different proportions. When folding a DNA triplex, the turn might be as tight as one that is devoid of nucleotides [176]. The telomeric duplex with repeated patterns cooperatively unfolds under the influence of forces without creating a triplex. A force ramp assay or a force jump experiment may produce a hopping characteristic or a stopping signal if there is a stabilizing triplex present. The remaining duplex area, which is around 17 bp long, may unfold before the triplex in this case. We saw hopping signals in addition to halting signals for all the mutants. A telomere sequence’s folding back triplex structures may hold together for an average of 3 s at a force of 20 pN. Telomeric triplexes may obstruct DNA cleavage and recombination, as well as force-regulated helicases [177,178] and force-sensitive RNA polymerases, due to their great mechanical stability and extended endurance [179]. Thus, two thymines significantly weaken the human telomeric triplex. This is further corroborated by the finding that AA mutation of TTAGGG to AAAGGG improved the chance of triplex formation in force-jump experiments by a factor of five. Our findings on GA TFOs revealed even greater triplex formation probabilities than those on AA mutants, indicating that DNA sequences significantly influence the likelihood that triplex structures will develop (Figure 4) (e.g., [175]).

## 3. Conclusions

The research on oligonucleotides with triplex-forming ability (TFO) in biological applications continues to profit tremendously from triplex technology. Even though the processes processing TFO-directed triplex formations and related lesions are not completely understood, it is known that proteins from numerous repair pathways are involved.

This repair method, as demonstrated by TFO targeted mutation generation, may be prone to error. Because of developments in the manufacture of chemically modified TFOs, we now have a better understanding of the cellular DNA repair mechanism, where proteins from distinct DNA repair pathways can work together or compete with one another in processing DNA lesions caused by TFOs. Triplex technology still has disadvantages, particularly for in vivo applications. These disadvantages include TFO absorption by cells, affinity and specificity for the binding site, and in vivo stability. As a result, current research is primarily focused on improving the efficacy of this approach of genome editing by chemical alterations to TFOs and improved cellular delivery mechanisms. Although there are still limits, triplex technology has played an important role in developing the science of targeted genome editing. While aptamers have been successfully integrated into pH-activatable systems and methods for altering aptamer binding capacities exist, the applications of designed pH-activatable aptamers remain largely unexplored. Aptamers that activate at weakly acidic pH might operate as an additional layer of control for the drug-delivery system as an extension of the drug-delivery systems mentioned above.
